# L-(+)-Ergothioneine ameliorates preeclampsia-associated vascular endothelial dysfunction by modulating the Nrf2-PPARγ-sFlt-1 axis

**DOI:** 10.1371/journal.pone.0358574

**Published:** 2026-09-18

**Authors:** Jingjin Gong, Yan Liu, Qingju Meng, Junwei Wu, Fang Chen, Yanwen Xu, Yanqiu Li, Qiwei Luo

**Affiliations:** 1 Obstetrics Department, Affiliated Hospital Group of Guangdong Medical University Panyu He Xian Memorial Hospital/Panyu Women and Children’s Medical Center, Guangdong Medical University (Guangzhou Panyu District Maternal and Child Health Hospital), Guangzhou, Guangdong, China; 2 Department of Biochemistry and Molecular Biology, School of Basic Medicine, Guangdong Medical University, Zhanjiang, Guangdong, China; Brigham and Women's Hospital, UNITED STATES OF AMERICA

## Abstract

**Background:**

Preeclampsia (PE) is a serious complication of pregnancy, with vascular endothelial dysfunction being a core pathological feature. This study aimed to investigate whether L-(+)-ergothioneine (LET) ameliorates PE-associated endothelial dysfunction by regulating the Nrf2-PPARγ-sFlt-1 axis.

**Methods:**

A LPS-induced trophoblast dysfunction model was established using lipopolysaccharide (LPS)-induced human trophoblast cells (HTR8/SVneo). Techniques including CCK-8 assay, flow cytometry, wound healing assay, ELISA, qPCR, Western blot, and immunofluorescence were employed to assess the effects of LET on cell viability, apoptosis, invasion, inflammatory cytokine levels, and the expression of key molecules in the signaling pathway.

**Results:**

LET significantly increased the viability of LPS-induced trophoblasts, promoted migration, inhibited apoptosis, and downregulated pro-inflammatory cytokines (IL-6, TNF-α, IFN-γ) and endothelial dysfunction markers (sFlt-1, ET-1, PAI-1), while upregulating the anti-inflammatory cytokine IL-4 and plasminogen activators (tPA, uPA). Mechanistically, LET inhibited Nrf2 nuclear translocation and promoted PPARγ expression, consequently reducing sFlt-1 levels. The protective effects of LET were mimicked by the PPARγ activator pioglitazone and reversed by the inhibitor FX-909. Furthermore, the supernatant from LET-treated trophoblasts promoted the viability and invasion of human umbilical vein endothelial cells (HUVECs).

**Conclusions:**

L-(+)-Ergothioneine improves trophoblast function and indirectly promotes endothelial recovery by modulating the Nrf2-PPARγ-sFlt-1 axis, suggesting a potential therapeutic target for preeclampsia.

## 1. Introduction

Preeclampsia (PE) is a pregnancy-specific hypertensive disorder that typically manifests after 20 weeks of gestation, posing a serious threat to maternal and fetal health. The pathogenesis of PE is complex, involving various physiological and pathological factors, among which vascular endothelial dysfunction is considered a primary pathological characteristic [1–3]. The incidence of PE is increasing worldwide, particularly in high-risk populations. Epidemiological data indicate that PE occurs in 3% to 8% of pregnancies, reaching up to 26% in some regions [4–6]. This condition not only increases the risk of maternal cardiovascular disease, renal insufficiency, and other complications but also adversely affects fetal growth and development, potentially leading to preterm birth, fetal growth restriction, and neonatal death [7,8].

Current research on PE focuses on its pathological mechanisms, early prediction, and intervention strategies. Existing studies have confirmed that oxidative stress, immune responses, and impaired vascular remodeling play key roles in the pathogenesis of PE [9–12]. Furthermore, Nrf2 (Nuclear factor erythroid 2-related factor 2) and PPARγ (Peroxisome proliferator-activated receptor gamma), as crucial transcription factors, play significant roles in regulating oxidative stress and inflammatory responses [13,14]. Although the functions of these factors in vascular regulation have been explored, their specific mechanisms in PE remain unclear, with conflicting results in the literature, necessitating further investigation.

Within this research field, while numerous studies have explored the mechanisms of PE, how modulation of the Nrf2-PPARγ and sFlt-1 (soluble fms-like tyrosine kinase-1) axis can improve vascular endothelial dysfunction in PE remains an inadequately addressed question [15,16]. The existing literature lacks in-depth research on the specific mechanism of L-(+)-ergothioneine (LET) in this process, which provides a strong rationale for this study. We aimed to investigate the regulatory role of LET to potentially offer new insights and approaches for clinical treatment.

This study focuses on the interactions between LET, Nrf2, PPARγ, and sFlt-1 and their impact on vascular endothelial function, aiming to elucidate their potential role in the pathophysiology of PE. Previous studies have shown that activation of Nrf2 and PPARγ can reduce oxidative stress levels, thereby improving endothelial function, which provides a theoretical basis for this research [17,18]. By investigating the interactions between these molecules, we hope to fill the current research gap and identify new targets for the prevention and treatment of PE.

To achieve these objectives, this study employed various research methods, including cell culture, molecular biology techniques, and biochemical analyses. The comprehensive application of these methods helped evaluate the regulatory effect of LET on the Nrf2-PPARγ-sFlt-1 axis at multiple levels, including molecular mechanisms, functional changes, and physiological effects. Specifically, we used cell-based experiments to assess the impact of LET on trophoblast and endothelial cell function and explored its mechanism of action. This multi-faceted research design ensures a comprehensive understanding of the research question and provides solid theoretical support for subsequent clinical applications.

In summary, this study aims to elucidate the potential regulatory role of LET in PE-associated vascular endothelial dysfunction, addressing a gap in current research. By thoroughly investigating the mechanism of the Nrf2-PPARγ-sFlt-1 axis, this study not only contributes to understanding the pathology of PE but may also provide new targeted strategies for future clinical treatment. This research is expected to offer new ideas and practical solutions for the prevention and treatment of PE, ultimately improving maternal and neonatal health.

## 2. Materials and methods

### 2.1 Materials

Lipopolysaccharide (LPS) from *Escherichia coli* was obtained from Sigma-Aldrich (Cat# L2880, St. Louis, MO, USA). L-(+)-ergothioneine (LET) was purchased from Sangon Biotech (Cat# A414843, Sangon, Shanghai, China). Pioglitazone (PIO, a PPARγ agonist) and FX-909 (a PPARγ antagonist) were sourced from MedChemExpress (Monmouth Junction, NJ, USA) with catalog numbers HY-13956 and HY-153344, respectively. For immunoblotting and immunofluorescence analyses, the following primary antibodies were utilized: anti-PPARγ (Catalog #2435, Cell Signaling Technology, Danvers, MA, USA), anti-Nrf2 (ab62352, Abcam, Cambridge, UK), and anti-GAPDH (Catalog #5174, Cell Signaling Technology).

### 2.2 HTR8/SVneo cell culture and passaging

Cells were cultured in a CO_2_ incubator (37°C, 5% CO_2_). When cell density reached 80–90%, cells were passaged. Cells were washed with PBS buffer, digested with 0.25% trypsin for 30 seconds, and the digestion was stopped with complete medium (89% RPMI-1640 + 10% FBS + 1% Penicillin/Streptomycin). The cell suspension was centrifuged at 300 g for 5 minutes, the supernatant was discarded, and the cell pellet was resuspended in fresh complete medium. Cells were passaged at a 1:3 ratio, seeded into T25 flasks, mixed gently by making a cross pattern, and placed back into the CO_2_ incubator for culture.

### 2.3 Establishment of PE cell model by LPS induction in HTR8/SVneo trophoblast cells

Concentration Gradient Experiment: Cells in the logarithmic growth phase were seeded into 96-well plates at 2.5 × 10^4^ cells/well (100 μL/well) and cultured overnight in a CO_2_ incubator. When cell density reached 60−70%, LPS solution was added at various concentrations (0.5, 2.5, 5, 10, 15, 20 μg/mL; n = 3 wells per group) for 24 hours [19]. IL-6 content in the cell supernatant was measured by ELISA (detailed protocol in Supplementary Information).

Time Gradient Experiment: Cells were seeded as above. At 60−70% confluence, LPS was added at concentrations of 0, 5, 10, 15 μg/mL (n = 3 wells per group) for 12, 24, or 36 hours. IL-6 content was measured by ELISA, and cell viability was assessed by CCK-8 assay (λ = 450 nm).

LET Concentration Gradient Experiment: (1) Cell seeding: Cells in the logarithmic growth phase were seeded into 24-well plates (3 replicates per concentration). (2) LPS induction and LET treatment: After 24 hours of culture (60−70% confluence), cells were treated with different concentrations of LET (250, 500, 750, 1000, 2000 ng/mL) for 24 hours. Cell viability was assessed by CCK-8 assay (λ = 450 nm).

### 2.4 Effect of LET on cell phenotype and the Nrf2/PPARγ-sFlt-1 axis

(1) Experimental Grouping: Cells in the logarithmic growth phase were seeded into 6-well plates at 3 × 10^5^ cells/well. Three groups were established: Control group, Model group, and LET group.
(2) Model Establishment and Drug Intervention: At 60–70% confluence, the Control group received complete medium, while the Model and LET groups were treated with complete medium containing 10 μg/mL LPS for 24 hours. The LET group then received 500 ng/mL LET solution for an additional 24 hours.
(3) Wound Healing Assay: After model establishment as in step 2, at 100% confluence, a wound was created using a 200 μL pipette tip guided by a ruler. Cells were gently washed twice with PBS. Images were taken at 0 h and 24 h after LET addition using a microscope.
(4) Apoptosis Assay: After interventions, cells were collected (2 million cells per group). 100 μL of apoptosis buffer and 5 μL of apoptosis staining reagent were added, followed by incubation in the dark at 4°C for 30 min, and then analysis by flow cytometry. Cell populations were gated using FSC vs SSC (Cell gate), then single cells were gated using FSC-H vs FSC-A (Single Cell gate). Apoptosis was analyzed using an Annexin V vs PI biparametric plot.
(5) Cell Viability Assay: After interventions, old medium was discarded, cells were washed twice with PBS, 100 μL of 10% CCK-8 solution (in basal medium) was added to each well, plates were gently shaken to mix, and incubated at 37°C in the dark for 30 min. Absorbance was measured at 450 nm.
(6) ELISA: After interventions, cell supernatants were collected. The levels of uPA, PAI-1, sFlt-1, tPA, ET-1, TNF-α, IL-6, IFN-γ, and IL-4 were measured according to kit instructions.
(7) PPARγ RNA and Protein Expression Levels: Detected by qPCR and Western blot (detailed protocols in Supplementary Information).

### 2.5 Effect of PPARγ activator and inhibitor on the Nrf2/PPARγ-sFlt-1 axis

(1) Experimental Grouping: Cells were seeded into 6-well plates as before. Five groups were established: Control, Model, LET, Pioglitazone (PIO), and FX-909 groups.
(2) Model Establishment and Drug Intervention: At 60–70% confluence, the Control group received complete medium, while other groups received medium containing 10 μg/mL LPS for 24 hours. Subsequently, the LET group received 500 ng/mL LET, the PIO group received 5 μg/mL PIO, and the FX-909 group received 0.5 μg/mL FX-909 (Note: text says PIO here, likely error, should be FX-909 inhibitor) for an additional 24 hours.
(3) Apoptosis Assay: Performed as described in 2.3.4.
(4) Cell Viability Assay: Performed as described in 2.3.5.
(5) ELISA: Supernatants were collected, and levels of sFlt-1, ET-1, and tPA were measured.
(6) Wound Healing Assay: Performed as described in 2.3.3, with images taken at 0 h and 24 h after drug addition.

### 2.6 Trophoblast-endothelial cell interaction

(1) HTR8/SVneo Cell Culture and Treatment: HTR8/SVneo cells in the logarithmic growth phase were seeded into 6-well plates at 3 × 10^5^ cells/well. Three groups: Group 1 (Control), Group 2 (Model), Group 3 (LET). At 60–70% confluence, Group 1 received complete medium, Groups 2 and 3 received medium with 10 μg/mL LPS for 24h. Group 3 then received 500 ng/mL LET for another 24h. Supernatants were collected, labeled as Supernatant 1, 2, and 3, filtered through a 0.45 μM sterile filter, and stored.
(2) Medium Preparation: Medium I: Supernatant 1 mixed with HUVEC-specific medium (5:1). Medium II: Supernatant 2 mixed with HUVEC-specific medium (5:1). Medium III: Supernatant 3 mixed with HUVEC-specific medium (5:1)
(3) HUVEC Experimental Grouping: HUVECs in the logarithmic growth phase were seeded into 6-well plates at 3 × 10^5^ cells/well. Three groups: Control, Model, LET groups.
(4) Intervention: At 60–70% confluence, the Control group received Medium I, the Model group received Medium II, and the LET group received Medium III, for 24 hours.
(5) Cell Viability Assay: Performed as described in 2.3.5 on HUVECs.
(6) Cell Invasion Assay (Transwell): HUVECs from step 4 were digested, centrifuged, and seeded into Transwell inserts at 2 × 10^5^ cells/well in 200 μL medium with 10% FBS. The lower chamber contained 600 μL medium with 20% FBS. Cells were cultured for 24h. Staining: After incubation, inserts were removed, liquid aspirated from the top chamber. Cells on the upper surface of the membrane were carefully wiped off with a custom-made cotton swab (partially removed head), followed by another moistened swab. Membranes were gently rinsed 3x with PBS, and residual PBS aspirated. 500 μL fixative (4% PFA) was added to the insert and 750 μL to the well, incubating for 20 min. Fixative was aspirated, 500 μL 0.1% crystal violet was added to the insert and 750 μL to the well, staining for 20 min. Crystal violet was removed, inserts were washed extensively with water, and liquid aspirated. Inserts were air-dried and photographed the next day.

### 2.7 Data processing

All experiments were performed at least three independent times (n = 3 biological replicates), with each condition tested in triplicate (technical replicates) unless otherwise specified. Data are presented as mean ± standard error of the mean (SEM). Statistical analyses were conducted using GraphPad Prism (version 9.0, GraphPad Software, San Diego, CA, USA). ImageJ (NIH, Bethesda, MD, USA) was used for wound healing area quantification, and FlowJo (version 10.8, BD Biosciences, Ashland, OR, USA) was used for flow cytometry data analysis.

**Fig 1:**
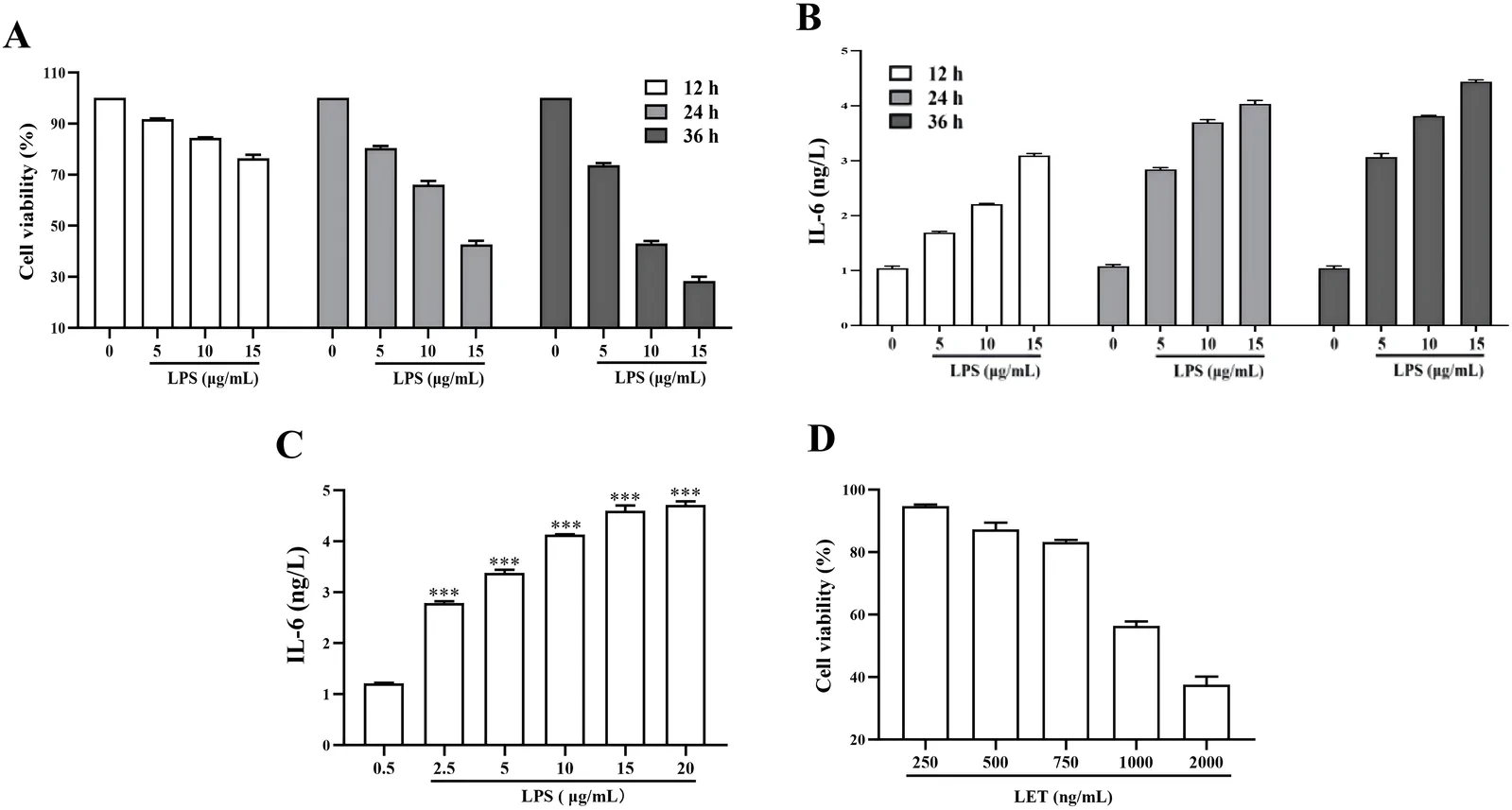
Effects of LPS and LET intervention on trophoblast cell viability and IL-6 levels under different concentrations and durations. (A) Cell viability and (B) IL-6 levels after intervention with LPS (0–15 μg/mL) for 12–36 h; (C) IL-6 levels after intervention with LPS (0.5–20 μg/mL) for 24 h; (D) Cell viability after intervention with LET (250–2000 ng/mL) for 24 h. Experiments were performed at least three independent times (n = 3 biological replicates), Statistical significance was set at *p* < 0.05. significance levels are indicated as follows: ***p < 0.001.

Normality of data distribution was assessed using the Shapiro–Wilk test, and homogeneity of variances was evaluated by Levene’s test. For comparisons between two groups, an unpaired two‑tailed Student’s t‑test was employed. For multiple group comparisons, one‑way analysis of variance (ANOVA) was performed, followed by Tukey’s honestly significant difference (HSD) post hoc test when comparing all groups, or Dunnett’s post hoc test when comparing treatment groups against a single control group. For experiments involving two independent factors (e.g., LPS concentration and treatment time in Fig 1), two‑way ANOVA followed by Bonferroni’s multiple comparisons test was applied.

Statistical significance was set at *p* < 0.05. In all figures, significance levels are indicated as follows: *p < 0.05, **p < 0.01, ***p < 0.001, and ****p < 0.0001; ns denotes not significant. Exact p‑values are provided in the corresponding figure legends where appropriate.

## 3. Results

### 3.1 Determination of LPS and LET intervention schemes for trophoblast cells

During the establishment of the LPS-induced trophoblast dysfunction model in trophoblast cells, to determine the optimal intervention concentration and duration of LPS, cells were first treated with 0, 5, 10, and 15 μg/mL LPS for 12, 24, and 36 hours. The results showed that cell viability decreased (Fig 1A) while IL-6 levels increased (Fig 1B) with rising LPS concentrations. Furthermore, prolonged intervention time led to reduced cell viability (Fig 1A) and increased IL-6 levels (Fig 1B). Based on these results, 24 hours was selected as the optimal intervention duration. Subsequently, to further determine the optimal intervention concentration, cells were treated with 0.5, 2.5, 5, 10, 15, and 20 μg/mL LPS for 24 hours, and the effect on IL-6 levels was measured. The results indicated that IL-6 levels increased with higher LPS concentrations (Fig 1C), leading to the final selection of 10 μg/mL as the optimal intervention concentration. To determine the optimal intervention concentration of L-(+)-Ergothioneine (LET), trophoblast cells were treated with 250, 500, 750, 1000, and 2000 ng/mL LET for 24 hours. The results demonstrated that cell viability decreased in a concentration-dependent manner with increasing LET concentrations (Fig 1D). Finally, based on the cell viability results, 500 nM was chosen as the LET intervention concentration, and 10 μg/mL LPS treatment for 24 hours was used to establish the PE trophoblast cell model.

### 3.2 Effect of LET on LPS-induced PE phenotype in trophoblasts

To investigate whether LET could ameliorate the LPS-induced PE-like phenotype in trophoblasts, HTR-8/SVneo cells were exposed to LPS in the presence or absence of 500 nM LET, after which a series of functional and biochemical assays were performed. We assessed cell viability, apoptosis, migration capacity, and the expression profiles of key inflammatory cytokines and endothelial regulatory factors.

**Fig 2:**
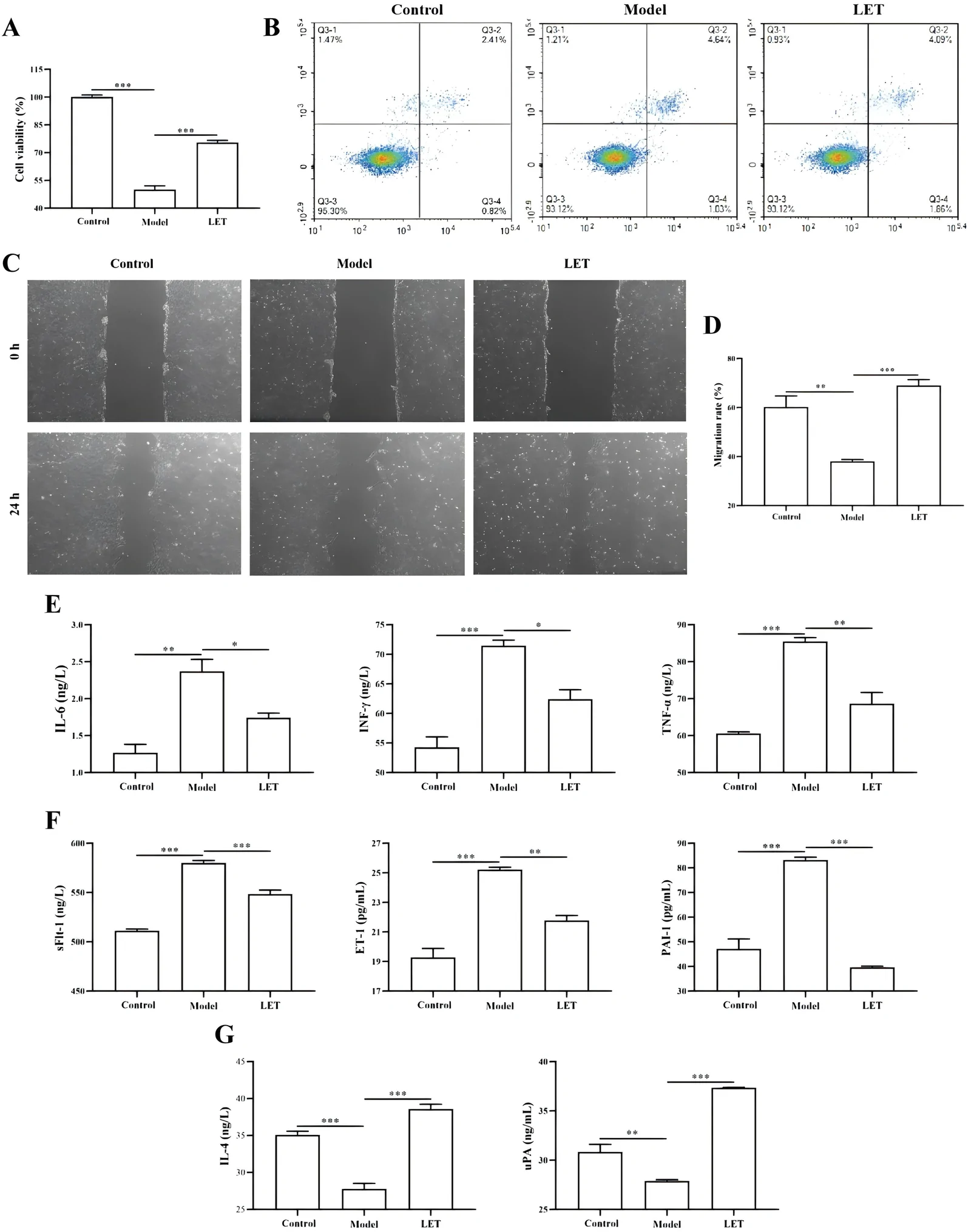
Effect of LET on LPS-induced PE phenotype in trophoblasts. (A) Cell viability after LET intervention; (B) Apoptosis detected by flow cytometry; (C) Representative images of wound healing assay at 0 h and 24 h; (D) Statistical analysis of wound healing; (E) Levels of pro-inflammatory factors (IL-6, TNF-α, IFN-γ); (F) Levels of endothelial dysfunction factors (sFlt-1, ET-1, PAI-1); (G) Levels of anti-inflammatory factor (IL-4) and plasminogen activators (tPA, uPA). Experiments were performed at least three independent times (n = 3 biological replicates), Statistical significance was set at *p* < 0.05. significance levels are indicated as follows: *p < 0.05, **p < 0.01 and ***p < 0.001.

The results demonstrated that LPS stimulation successfully induced a PE-like phenotypic shift in trophoblasts. Compared to the control group, the Model group exhibited a significant reduction in cell viability (Fig 2A), accompanied by a marked increase in apoptotic rate (Fig 2B). Wound healing assays further revealed that LPS significantly impaired trophoblast migratory capacity (Fig 2C and 2D). At the molecular level, LPS stimulation led to a pronounced upregulation of pro-inflammatory cytokines, including IL-6, TNF-α, and IFN-γ (Fig 2E). Additionally, the expression of endothelial dysfunction markers—sFlt-1, ET-1, and PAI-1—was significantly elevated (Fig 2F). Conversely, the levels of anti-inflammatory cytokine IL-4 and plasminogen activators tPA and uPA were markedly suppressed in the Model group (Fig 2G), collectively indicating a shift toward a pro-inflammatory, anti-angiogenic, and hypofibrinolytic state.

Treatment with LET effectively counteracted these LPS-induced alterations. Specifically, LET significantly restored trophoblast viability (Fig 2A) and promoted cell migration (Fig 2C and 2D). Although LET treatment reduced apoptosis, this effect was relatively modest and did not reach statistical significance compared to the Model group (Fig 2B). Notably, LET robustly suppressed the LPS-induced overexpression of pro-inflammatory cytokines (IL-6, TNF-α, IFN-γ) and endothelial dysfunction markers (sFlt-1, ET-1, PAI-1) (Fig 2E and 2F). Furthermore, LET treatment significantly enhanced the secretion of IL-4, tPA, and uPA (Fig 2G), restoring their levels toward those observed in the control group.

Taken together, these findings indicate that LET effectively mitigates LPS-induced trophoblast dysfunction by enhancing cell viability and migration, modulating the inflammatory response, and restoring the balance of endothelial regulators, thereby ameliorating key features of the PE-like phenotype.

### 3.3 Effect of LET on the Nrf2/PPARγ signaling pathway

**Fig 3:**
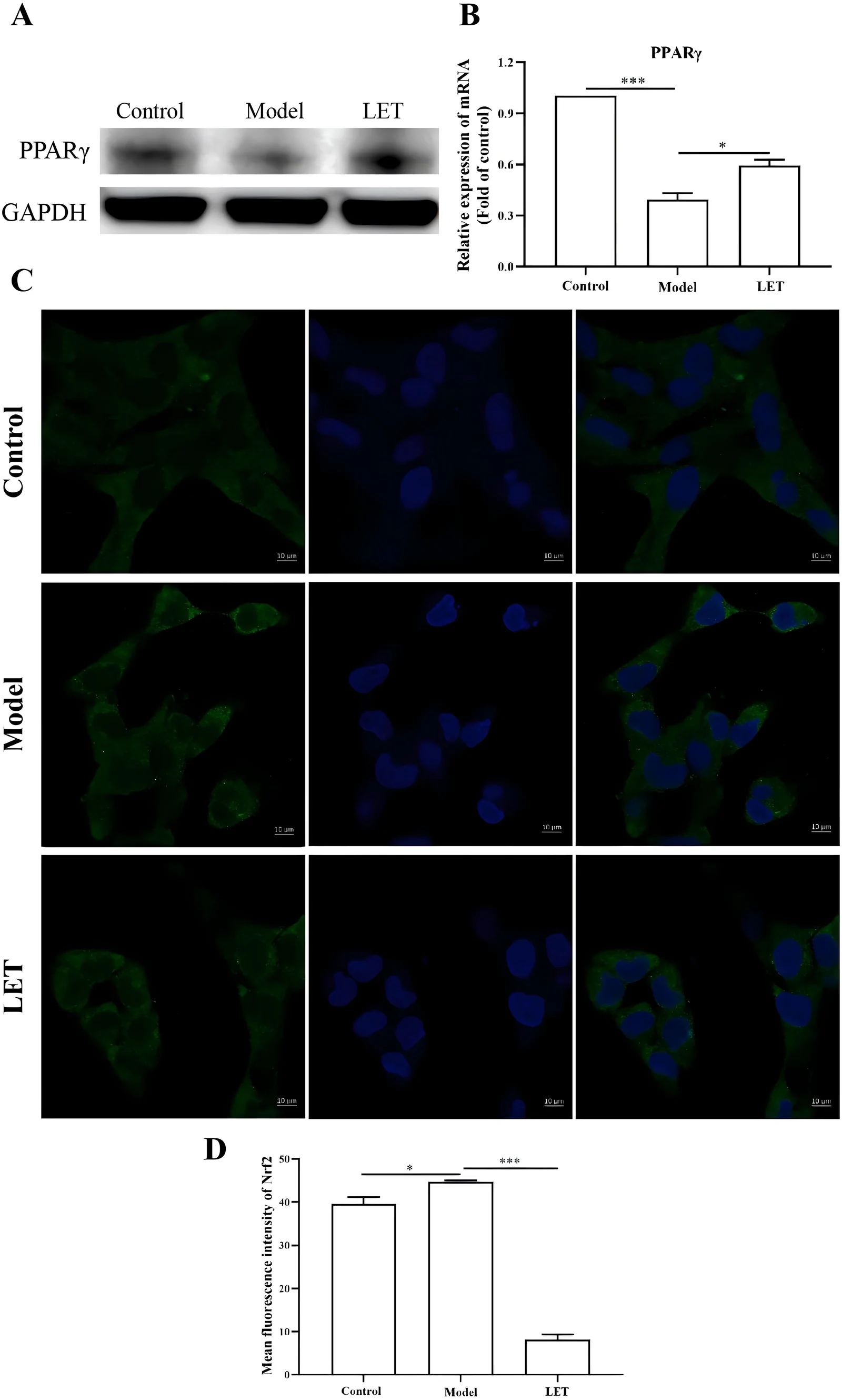
Regulatory effect of LET on the Nrf2/PPARγ signaling pathway. (A) WB analysis of PPARγ protein expression; (B) qPCR analysis of PPARγ mRNA expression; (C) Representative immunofluorescence images of Nrf2; (D) Statistical analysis of Nrf2 nuclear translocation. Experiments were performed at least three independent times (n = 3 biological replicates), Statistical significance was set at *p* < 0.05. significance levels are indicated as follows: *p < 0.05 and ***p < 0.001.

To further elucidate the molecular mechanisms underlying the protective effects of LET against the preeclampsia (PE)-like phenotype, we investigated its impact on the Nrf2/PPARγ signaling pathway, which has been implicated in the pathogenesis of PE. The protein expression levels of PPARγ were assessed by Western blotting, with GAPDH serving as the loading control, while its transcriptional levels were evaluated using quantitative real-time PCR (qPCR). Our results demonstrated that exposure to LPS significantly suppressed both PPARγ protein (Fig 3A) and mRNA (Fig 3B) expression. Notably, treatment with LET effectively reversed this LPS-induced downregulation, restoring PPARγ expression to levels comparable to the control group (Fig 3A and 3B).

Given that the transcriptional activity of PPARγ is closely associated with Nrf2, a key regulator of oxidative stress, we further examined the effect of LET on Nrf2 subcellular localization. Immunofluorescence staining revealed that under basal conditions, Nrf2 was predominantly localized in the cytoplasm. Upon LPS stimulation, a marked increase in Nrf2 nuclear translocation was observed (Fig 3C, quantified in 3D), indicating activation of the oxidative stress response. However, this translocation was significantly attenuated by LET treatment (Fig 3C and 3D), suggesting that LET modulates the Nrf2/PPARγ axis by suppressing Nrf2 nuclear accumulation. These findings indicate that LET may ameliorate PE-like pathological features by regulating the Nrf2/PPARγ signaling pathway, potentially through the inhibition of oxidative stress-driven Nrf2 activation.

### 3.4 LET alters PE phenotype and sFlt-1 levels via the Nrf2/PPARγ pathway

To further validate whether the protective effects of LET against the LPS-induced PE-like phenotype are mediated through the Nrf2/PPARγ signaling axis, we performed a series of pharmacological intervention experiments. An LPS-induced trophoblast dysfunction model was established and subsequently treated with either 5 μg/mL pioglitazone (PIO), a specific PPARγ agonist, or 0.5 μg/mL FX-909, a selective PPARγ antagonist, for 24 hours. We then evaluated key cellular functions, including cell viability, apoptosis, migration, and the secretion of critical endothelial regulatory factors.

**Fig 4:**
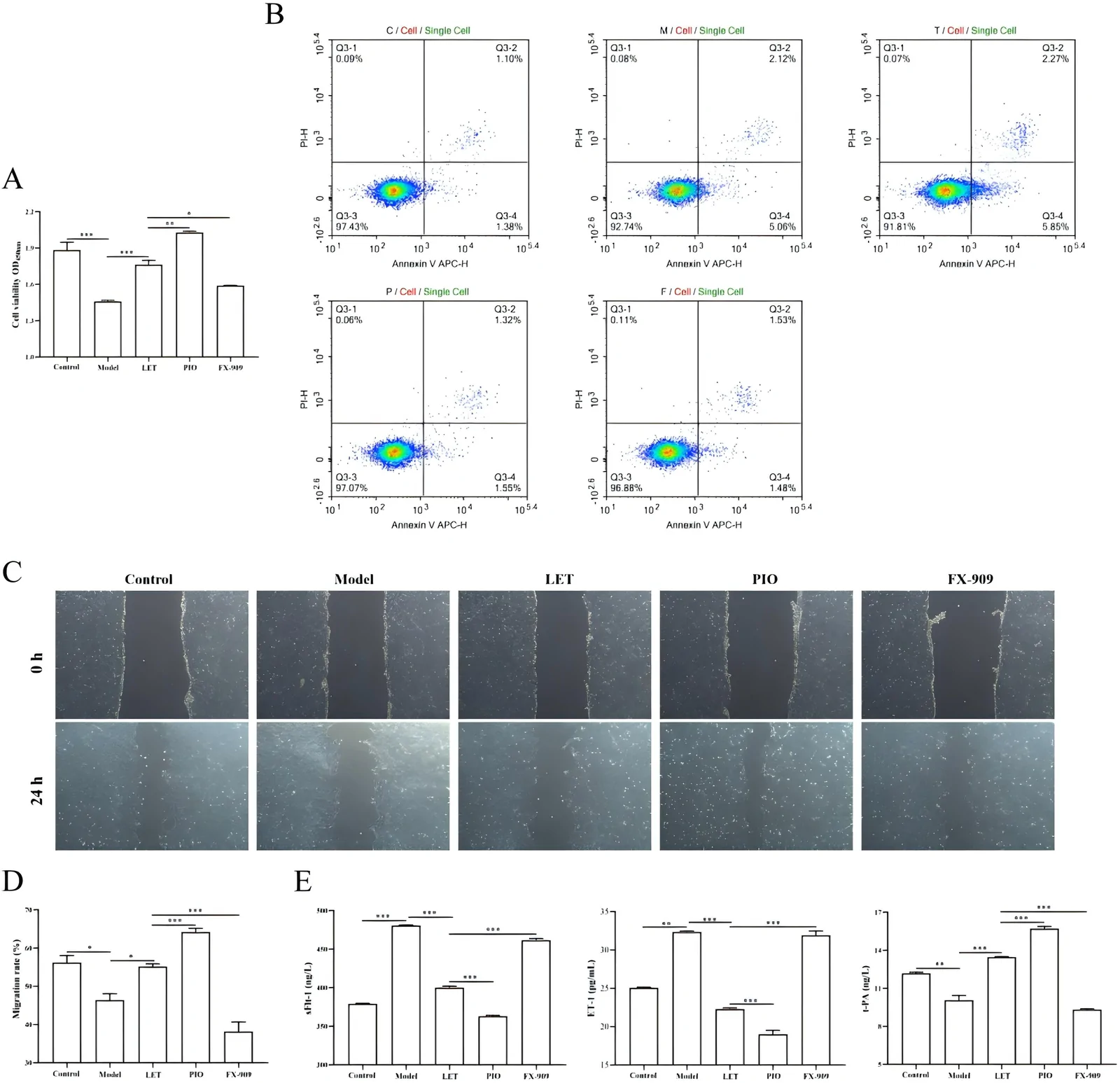
LET improves the trophoblast PE phenotype by activating the PPARγ pathway. (A) Cell viability; (B) Apoptosis detected by flow cytometry; (C) Representative wound healing images at 0 h and 24 h; (D) Statistical analysis of wound healing; (E) Levels of sFlt-1, ET-1, and tPA. Experiments were performed at least three independent times (n = 3 biological replicates), Statistical significance was set at *p* < 0.05. significance levels are indicated as follows: *p < 0.05, **p < 0.01 and ***p < 0.001.

The results demonstrated that activation of PPARγ by PIO mimicked the beneficial effects of LET. Compared to the LPS-only group, PIO treatment significantly enhanced trophoblast viability (Fig 4A) and migratory capacity (as assessed by wound healing or Transwell assays, Fig 4C and 4D), while concurrently reducing the rate of apoptosis (Fig 4B). Furthermore, PIO treatment rectified the imbalance of angiogenic regulators induced by LPS, as evidenced by decreased secretion of the anti-angiogenic factors sFlt-1 and ET-1, alongside a marked increase in the pro-fibrinolytic factor tPA (Fig 4E).

In stark contrast, pharmacological inhibition of PPARγ with FX-909 exacerbated the PE-like phenotype. Cells treated with FX-909 exhibited a further decline in cell viability (Fig 4A) and migration (Fig 4C and 4D) compared to the LPS group. Consistently, FX-909 treatment led to a significant increase in the secretion of sFlt-1 and ET-1, while further suppressing tPA levels (Fig 4E).

Collectively, these results provide functional evidence that LET activates the PPARγ pathway. By doing so, it counteracts LPS-induced trophoblast dysfunction—enhancing viability and migration, reducing apoptosis, and restoring the balance of endothelial regulators—thereby ameliorating the PE-like pathological phenotype.

### 3.5 LET Improves endothelial function via trophoblast-secreted factors

Given that trophoblast dysfunction and the subsequent imbalance of angiogenic regulators (such as sFlt-1) critically impact the maternal endothelium in PE pathogenesis, we next investigated whether the protective effects of LET on trophoblasts could indirectly influence endothelial function via paracrine signaling. To test this, we collected conditioned media from trophoblasts subjected to different treatments (Control, LPS Model, and LPS + LET) and applied them to human umbilical vein endothelial cells (HUVECs) for 24 hours. Endothelial cell viability and migratory capacity were then assessed.

**Fig 5:**
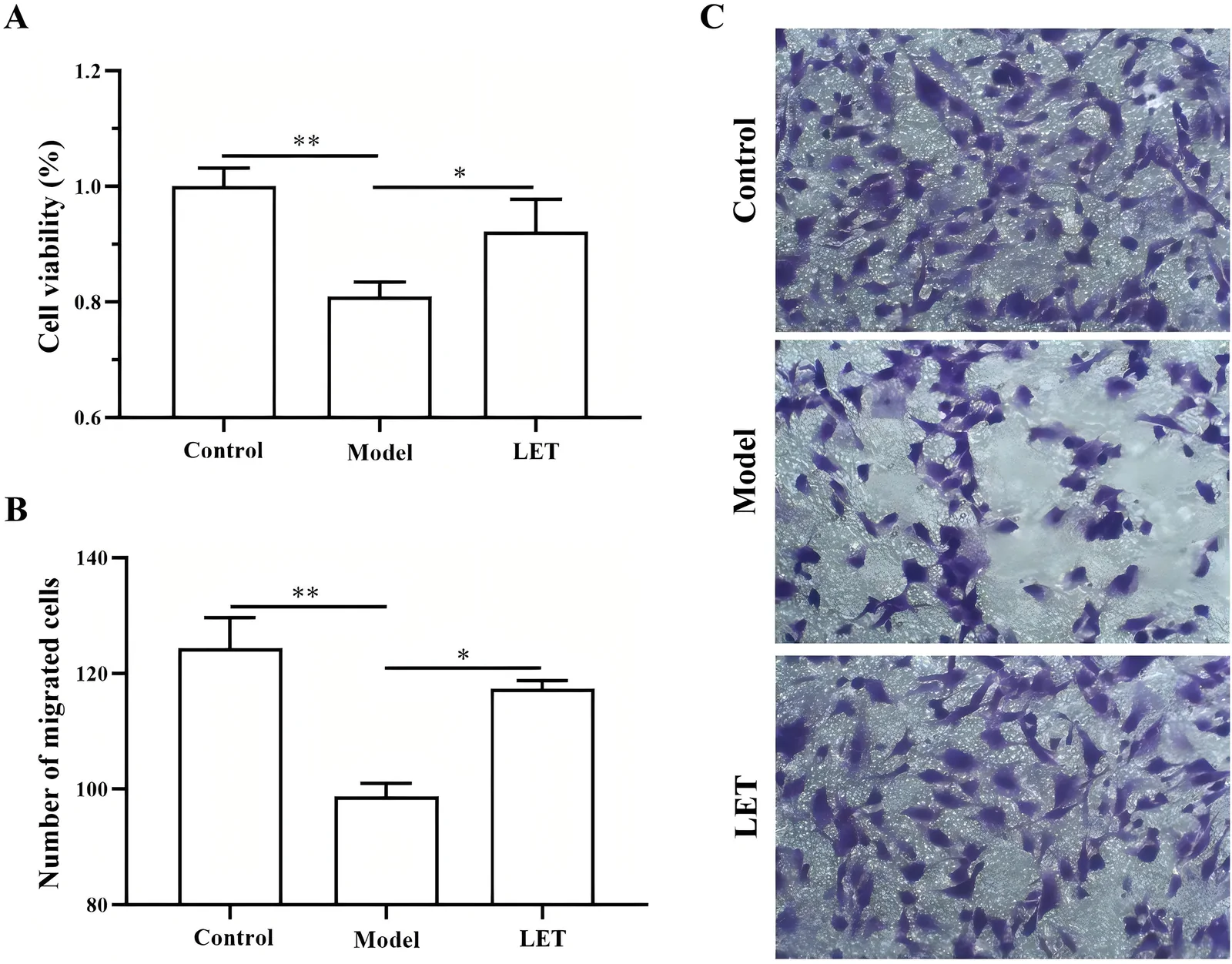
Supernatant from LET-treated trophoblasts promotes endothelial cell functional recovery. (A) HUVEC viability; (B) Number of migrated endothelial cells; (C) Representative images of endothelial cell migration (Transwell assay). Experiments were performed at least three independent times (n = 3 biological replicates), Statistical significance was set at *p* < 0.05. significance levels are indicated as follows: *p < 0.05 and **p < 0.01.

As shown in Fig 5A, exposure to conditioned media from LPS-treated trophoblasts (Model group) significantly reduced HUVEC viability compared to the Control group. Furthermore, wound healing assays revealed that this conditioned media markedly impaired HUVEC migration (Fig 5B and 5C), indicating that LPS-induced trophoblasts secrete factors that compromise endothelial function.

In contrast, conditioned media from trophoblasts co-treated with LET (LET group) effectively reversed these detrimental effects. HUVECs cultured with LET group supernatant exhibited significantly restored cell viability (Fig 5A) and enhanced migratory capacity (Fig 5B and 5C), comparable to levels observed in the Control group.

These findings suggest that LET treatment reprograms the secretory profile of trophoblasts—likely by reducing anti-angiogenic factors such as sFlt-1 and ET-1 while promoting pro-angiogenic and fibrinolytic factors—thereby creating a paracrine environment that supports endothelial cell survival and migration. This trophoblast-endothelial crosstalk may represent a key mechanism through which LET exerts its therapeutic effects in mitigating the vascular dysfunction characteristic of preeclampsia.

## 4. Discussion

Preeclampsia, a serious obstetric complication, involves complex pathogenesis where vascular endothelial dysfunction is a core element [20,21]. This study focused on the mechanism by which L-(+)-ergothioneine (LET) protects trophoblast function and vascular endothelium in PE, systematically elucidating, for the first time, the potential pathway through which LET ameliorates PE pathological phenotypes via the Nrf2-PPARγ-sFlt-1 axis.

In the present study, we established an in vitro model of trophoblast dysfunction by stimulating HTR-8/SVneo cells with lipopolysaccharide (LPS). This model successfully recapitulated key pathological features associated with preeclampsia (PE), including reduced cell viability, increased apoptosis, impaired migratory capacity, and dysregulated expression of inflammatory cytokines and vasoactive factors. Treatment with LET significantly ameliorated these phenotypic alterations. Notably, LET not only suppressed the release of pro-inflammatory cytokines such as IL-6, TNF-α, and IFN-γ but also exerted multifaceted regulatory effects on factors closely linked to endothelial dysfunction. Specifically, LET reduced the secretion of anti-angiogenic and vasoconstrictive factors (sFlt-1, ET-1, PAI-1) while enhancing the expression of fibrinolytic and vasoprotective factors (tPA and uPA). These findings suggest that LET may improve the placental microenvironment and systemic endothelial function through multi-target regulation, offering a potential therapeutic strategy for PE.

However, it is important to acknowledge the limitations of the cellular model employed in this study. The HTR-8/SVneo cell line, while widely used as a surrogate for extravillous trophoblasts (EVTs), has been shown by global transcriptomic analyses to differ substantially from primary human placental tissue and sorted trophoblast subtypes [22,23]. Furthermore, HTR-8/SVneo cultures are known to consist of a heterogeneous mixture of epithelial (trophoblast-like) and mesenchymal/stromal cells, which may introduce variability and limit the interpretability of results [24,25]. As such, HTR-8/SVneo may not represent the optimal in vitro model currently available, and findings derived from this line should be interpreted with caution.

To address these limitations and strengthen the translational relevance of our findings, future studies should aim to validate key observations in more physiologically relevant systems. These may include primary human trophoblasts or human trophoblast stem cells (hTSCs) derived from placental tissues, which more faithfully recapitulate the transcriptional and functional characteristics of authentic trophoblast subtypes [26]. Additionally, incorporating three-dimensional organoid cultures or placental explant models could provide further insights into the multicellular interactions underlying PE pathogenesis and the therapeutic potential of LET. Despite the current limitations, our results provide a foundation for understanding the mechanistic actions of LET and warrant further investigation in more advanced preclinical models.

Mechanistically, this study demonstrated that LET inhibits Nrf2 nuclear translocation and promotes PPARγ transcription and expression, consequently regulating downstream sFlt-1 production. Nrf2, a key regulator of oxidative stress, is often aberrantly activated in PE placental tissue. Although its early activation offers some cytoprotection, sustained high activity may exacerbate inflammatory responses and impair trophoblast function. In this study, LPS activated Nrf2 nuclear translocation, which was significantly inhibited by LET intervention, suggesting LET may mitigate excessive inflammation caused by oxidative stress via negative feedback regulation. On the other hand, PPARγ, a nuclear receptor family member, possesses anti-inflammatory, antioxidant, and pro-differentiation functions [27,28]. Our results showed that LET reversed the LPS-induced downregulation of PPARγ expression. Validation experiments using the pharmacological agonist pioglitazone and inhibitor FX-909 further confirmed that PPARγ activation is a crucial mechanism for LET’s protective effects. Importantly, PPARγ regulates the expression of downstream sFlt-1, providing a new perspective for understanding angiogenic imbalance in PE.

Furthermore, using a trophoblast-endothelial cell interaction model, we found that supernatant from LET-treated trophoblasts significantly improved HUVEC viability and migration capacity, indicating that the LET-modulated trophoblast secretome exerts paracrine effects that indirectly promote endothelial functional recovery. This suggests that LET not only acts directly on placental cells but may also influence the maternal vascular system by modulating placental-derived factors, highlighting its therapeutic potential through multi-cellular and multi-pathway synergistic actions [29,30].

Despite the promising findings obtained from our in vitro experiments, several limitations of this study should be acknowledged. Most notably, the current investigation was conducted exclusively using cellular models and lacks validation in experimental animal models of preeclampsia. While HTR-8/SVneo cells and HUVECs provide valuable insights into trophoblast and endothelial cell biology, they cannot fully recapitulate the complex in vivo physiological environment, including hemodynamic changes, immune cell interactions, and multi-organ crosstalk that characterize PE pathogenesis. Animal models, such as the LPS-induced or reduced uterine perfusion pressure (RUPP) rat models of PE, would offer a more holistic platform to evaluate the therapeutic efficacy of LET and validate the proposed Nrf2-PPARγ-sFlt-1 signaling axis under physiologically relevant conditions. Such in vivo studies would also enable assessment of critical parameters including blood pressure regulation, proteinuria, placental morphology, and fetal outcomes, which are essential endpoints for evaluating potential PE therapeutics. Future investigations incorporating appropriate animal models are therefore warranted to substantiate our current findings and advance the translational potential of LET for PE treatment.

This study has an inherent limitation, namely that the conditioned medium inevitably contains residual therapeutic agents. To maximize the retention of active factors secreted during treatment, we did not include a washing step when collecting the supernatant of HTR8/SVneo cells. As a result, the culture medium applied to HUVECs still contained relatively high concentrations of LPS (theoretical diluted concentration ~1.67 μg/mL) and LET. Although our data clearly show that conditioned media from the LPS-treated group and the LPS + LET rescue group exerted distinct biological effects on HUVECs, reflecting the remodeling of the trophoblast microenvironment following LET intervention, we cannot completely rule out the possibility that residual LPS or LET directly acts on HUVECs. Notably, as a potent endotoxin, LPS may exist in the culture medium in protein-bound or vesicle-encapsulated forms, and its bioactivity under such conditions may differ from that of its free form. This complexity makes it difficult for simple “drug-added controls” to fully simulate the real conditions. Future studies should employ ultrafiltration or dialysis with a molecular weight cutoff of 10 kDa to physically separate the large-molecule secretome from small-molecule drug residues in the conditioned medium, thereby precisely delineating the respective contributions of trophoblast-derived factors and residual therapeutic agents to the regulation of endothelial cells.

## 5. Conclusion

In conclusion, this study demonstrate that LET ameliorates PE-associated cellular dysfunction by modulating the Nrf2-PPARγ-sFlt-1 axis, exhibiting significant anti-inflammatory, antioxidant, and vascular protective effects. These results not only deepen the understanding of PE pathogenesis but also provide a theoretical basis and experimental support for developing LET or similar active molecules as intervention strategies. Future research should build on these findings by conducting *in vivo* animal experiments and clinical sample validation to facilitate the translation of this compound towards clinical application.

## Supporting information

S1 File Supplementary materials and methods and tables. This file provides comprehensive details on the experimental procedures, including ELISA for IL-6 detection, PPARγ gene expression analysis via qPCR, Western blotting for PPARγ protein detection, and immunofluorescence assessment of Nrf2 nuclear localization. Additionally, it contains the following supporting tables: Table S1. Reverse transcription cDNA reaction setup. Table S2. Gene primer sequences. Table S3. qPCR reaction setup. Table S4. qPCR reaction conditions.
(DOCX)

S1 Fig GAPDH. (TIF)

S2 Fig PPAR. (TIF)
