# Supplementary material for: L-(+)-Ergothioneine ameliorates preeclampsia-associated vascular endothelial dysfunction by modulating the Nrf2-PPARγ-sFlt-1 axis
Source: PLoS One. 2026 Sep 18;21(9):e0358574. doi: 10.1371/journal.pone.0358574 (PMC13588364; doi:10.1371/journal.pone.0358574)
Supplement: Supplementary file 1 [file pone.0358574.s001.docx]

**Supporting Information**

**L-(+)-Ergothioneine Ameliorates Preeclampsia-Associated Vascular Endothelial Dysfunction by Modulating the Nrf2-PPARγ-sFlt-1 Axis**

Jingjin Gong^1,^†, Yan Liu^2,^†, Qingju Meng^1^, Junwei Wu^1^, Fang Chen^1^, Yanwen Xu^1^, Yanqiu Li^1,^*, Qiwei Luo^1,^*

^1^Obstetrics Department, Affiliated Hospital Group of Guangdong Medical University Panyu He Xian Memorial Hospital/Panyu Women and Children's Medical Center, Guangdong Medical University (Guangzhou Panyu District Maternal and Child Health Hospital), 511400 Guangzhou, Guangdong, China

^2^Department of Biochemistry and Molecular Biology, School of Basic Medicine, Guangdong Medical University, 524023 Zhanjiang, Guangdong, China

*Corresponding author E-mail: liyanqiu1977@163.com (Yanqiu Li); 3251627824@qq.com (Yiwei Luo).

†These authors contributed equally.

**Materials and Methods**

*ELISA for IL-6 Detection*

(1) **Standard Curve Preparation:** The standard was diluted sequentially according to the following table:

| **Concentration** | **Procedure** |
| --- | --- |
| 20 ng/L | Add 150 μL of the stock standard to 150 μL of standard diluent |
| 10 ng/L | Add 150 μL of the 20 ng/L standard to 150 μL of standard diluent |
| 5 ng/L | Add 150 μL of the 10 ng/L standard to 150 μL of standard diluent |
| 2.5 ng/L | Add 150 μL of the 5 ng/L standard to 150 μL of standard diluent |
| 1.25 ng/L | Add 150 μL of the 2.5 ng/L standard to 150 μL of standard diluent |

(2) **Loading:** Wells were designated as follows: blank wells (no sample or enzyme conjugate), standard wells, and test sample wells, with three replicate wells per group. Add 50 μL of standard per well to the antibody-coated microplate. Add 40 μL of sample diluent and 10 μL of test sample to the sample wells. Avoid touching the well walls during loading and mix gently.

(3) **Incubation:** Seal the plate with a sealing film and incubate at 37°C for 30 minutes.

(4) **Solution Preparation:** Dilute the 20 × concentrated wash buffer 20-fold with distilled water for later use.

(5) **Washing:** Carefully remove the sealing film, discard the liquid, and blot dry. Fill each well completely with wash buffer, let stand for 30 seconds, then discard. Repeat this process 5 times and blot dry.

(6) **Enzyme Addition:** Add 50 μL of enzyme conjugate to each well, except the blank wells. Incubate at 37°C for 30 minutes.

(7) **Washing:** Repeat the washing process as described in step 5.

(8) **Color Development:** Add 50 μL of Substrate A to each well, followed by 50 μL of Substrate B. Gently shake to mix well. Incubate at 37°C protected from light for 20 minutes.

(9) **Stop Reaction:** Add 50 μL of stop solution to each well to terminate the reaction.

(10) **Measurement:** Measure the absorbance at 450 nm (ensure measurement is completed within 15 minutes after adding the stop solution). Use the blank wells for OD calibration.

*PPARγ Gene Detection*

(1) **Total RNA Extraction:** After the experiment, cells were collected. Add 1 mL of RNA extraction reagent to the cell pellet, gently vortex or pipette to lyse cells. Add 100 μL of chloroform substitute, invert the tube vigorously for 15 sec, mix thoroughly, and let stand for 3 min. Centrifuge at 12,000 rpm for 10 min at 4 °C. Transfer 400 μL of the upper aqueous phase to a new tube. Add 550 μL of isopropanol, invert to mix. Place at -20 °C for 15 min. Centrifuge at 12,000 rpm for 10 min at 4 °C; the white precipitate at the bottom of the tube is RNA. Carefully aspirate the supernatant. Add 1 mL of 75% ethanol, invert to wash the pellet. Centrifuge at 12,000 rpm for 5 min at 4 °C. Repeat the wash step once. Aspirate the liquid completely and air-dry for 3-5 min. Dissolve the RNA in 15 μL of RNase-free water. Determine RNA concentration and purity using a Nanodrop 2000: blank the instrument, then apply 2.5 μL of RNA sample to the pedestal, lower the sample arm, and initiate absorbance detection using the software. Dilute RNA samples with excessively high concentrations appropriately to a final concentration of 200 ng/μL for subsequent reverse transcription.

(2) **Reverse Transcription:** Prepare the reaction mixture (recommended 20 μL system, Reverse Transcription Kit, Cat. No. G3337) as shown in **Table S1**:

**Table S1**. Reverse Transcription cDNA Reaction Setup

| **Reagent** | **Volume** | **Unit** |
| --- | --- | --- |
| 5×SweScript All-in-One SuperMix for qPCR | 4 | µL |
| gDNA Remover | 1 | µL |
| Total RNA * | 10 | µL |
| RNase free water | Added to 20 | µL |

Place the 20 μL reaction mixture in a PCR machine, set the volume to 20 μL, and run the following program: Stage 1: 25°C for 5 min; Stage 2: 42°C for 30 min; Stage 3: 85°C for 5 sec. Start the instrument to perform reverse transcription. After completion, the resulting cDNA was used for subsequent experiments.

(3) **Quantitative PCR (qPCR):** The reaction mixture was prepared using the ChamQ Universal SYBR qPCR Master Mix kit. The relative expression level of the target gene was calculated using the 2^(-ΔΔCt) method based on the Ct values of the target gene and the internal reference gene (β-actin/GAPDH), and the data were processed accordingly. Gene primer sequences are listed in **Table S2**.

**Table S2**. Gene Primer Sequences

| **Gene** | **Forward Primer** | **Reverse Primer** |
| --- | --- | --- |
| *GAPDH* | GGAAGCTTGTCATCAATGGAAATC | AAGGAAGGCTGGAAGAGTGC |
| *PPARγ* | AGACCACTCCCACTCCTTTGAT | GAGATGCAGGCTCCACTTTGAT |

Using a 0.1 mL PCR plate, prepare the reaction mixture according to **Table S3**. Prepare three tubes for each reverse transcription product. After loading, seal the plate using a PCR sealing film and a plate sealer, and centrifuge briefly in a microplate centrifuge.

**Table S3**. qPCR Reaction Setup

| **Reagent** | **Volume** | **Unit** |
| --- | --- | --- |
| cDNA | 2.00 | µL |
| Forward Primer | 0.75 | µL |
| Reverse Primer | 0.75 | µL |
| 2×Universal Blue SYBR Green qPCR Master Mix | 7.50 | µL |
| dd H_2_O | 4.00 | µL |
| Total | 15.00 | µL |

The qPCR reaction conditions were set according to **Table S4**:

**Table S4**. qPCR Reaction Conditions

| **Stage** | **Process** | **Cycles** | **Temperature** | **Time** |
| --- | --- | --- | --- | --- |
| 1 | Pre-denaturation | 1 | 95℃ | 30sec |
| 2 | Amplification | 40 | 95℃ | 15sec |
|  |  |  | 60℃ | 30sec |
| 3 | Melt Curve | Instrument default melt curve acquisition | | |

The reaction volume was 20 μL. Samples were placed in the PCR instrument, and the qPCR run was initiated. Data were exported after completion for analysis.

*Western Blot Analysis (PPARγ Protein Detection)*

(1) **Total Protein Extraction:** After the experiment, cells were collected.

A. **Lysis Buffer Preparation:** Thaw the lysis buffer and mix well. Shortly before use, add the appropriate ratio of protease or phosphatase inhibitors to the required volume of lysis buffer, mix thoroughly.

B. **Sample Lysis:** Add 100-200 μL of lysis buffer per well of a 6-well plate. Pipette several times to ensure complete contact between the lysis buffer and cells.

C. **Protein Collection:** After complete lysis, centrifuge at 12,000 rpm for 5 min at 4 °C, and collect the supernatant.

D. **Protein Denaturation:** Mix the protein solution with loading buffer at an appropriate ratio, boil for 10 min, allow to cool slowly to room temperature, centrifuge briefly, and store at -20°C.

(2) **BCA Protein Concentration Assay:**

A. **BCA Working Solution Preparation:** Calculate the total volume of working solution required. According to the calculated volume, prepare the BCA working solution by mixing Reagent A and Reagent B at a 50:1 volume ratio, and mix thoroughly.

B. **Protein Concentration Measurement:** Add 20 μL of each diluted BSA standard and 20 μL of each test sample to the microplate wells. Add 200 μL of working solution to each well and mix thoroughly. Cover the plate and incubate at 37°C for 30 min. Measure the absorbance at 562 nm (A562) for each sample and BSA standard using a microplate reader. Plot the standard curve and calculate the protein concentration of the samples.

(3) **SDS-PAGE Electrophoresis:**

Prepare 1× MOPS-SDS Running Buffer. Assemble the pre-cast gel. Add sufficient MOPS-SDS Running Buffer to the electrophoresis tank. Before loading, rinse all wells with running buffer. Load the denatured protein samples into the wells. Run the gel at 160V until the bromophenol blue dye front just migrates out of the gel.

(4) **Membrane Transfer (Wet Transfer):**

Cut filter paper and PVDF membrane to appropriate sizes. Soak the filter paper and sponges in transfer buffer until fully saturated, equilibrate for 5 min. Activate the PVDF membrane in 100% methanol for 30 sec, then soak in transfer buffer until fully saturated, equilibrate for 5 min. Rinse the gel in ultrapure water for 2 min to remove surface SDS; then equilibrate the gel in transfer buffer. Assemble the transfer sandwich in the following order (from cathode to anode): cathode pad, filter paper, PVDF membrane, gel, filter paper, anode pad. Remove all air bubbles carefully during assembly. Place the sandwich into the transfer tank filled with transfer buffer. Transfer at a constant current of 375 mA for 40 min under cold conditions (using a cooling unit or in a cold room).

(5) **Immunoblotting:**

A. **Blocking:** Block the membrane with 5% skim milk solution at room temperature for 1 h.

B. **Primary Antibody Incubation:** Incubate the membrane with the appropriately diluted primary antibody at 4°C overnight.

C. **Washing:** Wash the membrane with TBST 5 times, 5 min each.

D. **Secondary Antibody Incubation:** Incubate the membrane with the appropriately diluted HRP-conjugated secondary antibody at room temperature for 1 h.

E. **Washing:** Wash the membrane with TBST 5 times, 5 min each.

F. **Detection:** Prepare ECL working solution: Mix equal volumes of ECL Solution A and B in a clean tube (use separate tips to avoid cross-contamination). Apply the ECL working solution evenly over the membrane. Place the membrane in a chemiluminescence imaging system and detect the signals according to the instrument's instructions.

*Nrf2 Nuclear Localization (Immunofluorescence)*

After interventions, discard the culture medium, wash cells twice with PBS, and fix with 4% paraformaldehyde for 30 min at room temperature. Transfer the coverslips (cell side up) to a new plate and wash with PBS (gentle shaking suffices). Permeabilize cells with 0.3% Triton X-100 in PBS for 15-20 min at room temperature, then wash with PBS. Block with 5% BSA in PBS for 15-20 min at room temperature, then wash with PBS. Incubate with primary antibody diluted in BSA solution at 4°C overnight. Wash three times with PBS, 2 min each soak. Incubate with fluorescently labeled secondary antibody diluted in PBS, protected from light, for 1 h at room temperature. Wash three times with PBS, 2 min each soak. Mount the coverslips: Pre-warm the DAPI/mounting medium. Place 6-10 μL on a glass slide. Carefully remove the coverslip from the well, blot excess liquid gently, and place it cell-side down onto the mounting medium on the slide. Store the mounted slides protected from light at 4°C. Acquire images using a 63× objective lens. Select the 488 nm channel (for FITC/AF488) and the DAPI channel. Acquire 5 fields of view per sample.
